# Determinants of effective treatment coverage for major depressive disorder in the WHO World Mental Health Surveys

**DOI:** 10.1186/s13033-022-00539-6

**Published:** 2022-06-23

**Authors:** Daniel V. Vigo, Alan E. Kazdin, Nancy A. Sampson, Irving Hwang, Jordi Alonso, Laura Helena Andrade, Olatunde Ayinde, Guilherme Borges, Ronny Bruffaerts, Brendan Bunting, Giovanni de Girolamo, Silvia Florescu, Oye Gureje, Josep Maria Haro, Meredith G. Harris, Elie G. Karam, Georges Karam, Viviane Kovess-Masfety, Sing Lee, Fernando Navarro-Mateu, José Posada-Villa, Kate Scott, Juan Carlos Stagnaro, Margreet ten Have, Chi-Shin Wu, Miguel Xavier, Ronald C. Kessler

**Affiliations:** 1 Department of Psychiatry, University of British Columbia, UBC Hospital – Detwiller Pavilion, Room 2813, 2255 Wesbrook Mall, UBC Vancouver Campus, Vancouver, BC V6T 2A1 Canada; 2 Department of Global Health and Social Medicine, Harvard Medical School, Boston, MA USA; 3 Department of Psychology, Yale University, 2 Hillhouse Avenue- 208205, New Haven, CT 06520 USA; 4 Department of Health Care Policy, Harvard Medical School, 180 Longwood Avenue, Boston, MA 02115 USA; 5 IMIM-Hospital del Mar Medical Research Institute, PRBB Building, Doctor Aiguader, 88, 08003 Barcelona, Spain; 6 University of São Paulo Medical School, Núcleo de Epidemiologia Psiquiátrica - LIM 23, Rua Dr. Ovidio Pires de Campos, 785, São Paulo, CEP 05403-010 Brazil; 7 Department of Psychiatry, University College Hospital, University of Ibadan, Ibadan, 5116 PMB Nigeria; 8 National Institute of Psychiatry Ramón de La Fuente Muñiz, Calzada México-Xochimilco, 101, Colonia San Lorenzo Huipulco, 14370 Mexico City, DF Mexico; 9 University Psychiatric Center, University of Louvain – KU Leuven, Herestraat 49, B-3000 Leuven, Belgium; 10 School of Psychology, Ulster University, College Avenue, Londonderry, BT48 7JL UK; 11 Istituti di Ricovero e Cura a Carattere Scientifico (IRCCS)-St. John of God Clinical Research Centre, Via Pilastroni 4, Brescia, Italy; 12 National School of Public Health, Management and Development, 31 Vaselor Str., 21253 Bucharest, Romania; 13 Parc Sanitari Sant Joan de Déu, Dr. Antoni Pujades, 42, 08830 Sant Boi de Llobregat, Barcelona, Spain; 14 School of Public Health, The University of Queensland, c/o QCMHR, Locked Bag 500, Archerfield, QLD 4108 Australia; 15 Queensland Centre for Mental Health Research, The Park Centre for Mental Health, Wolston Park Rd,, Wacol, QLD 4076 Australia; 16 Department of Psychiatry & Clinical Psychology, St. George Hospital University Medical Center, Beirut, 166378 Ashrafieh Lebanon; 17 Department of Psychiatry and Clinical Psychology, Faculty of Medicine, Balamand University, Rond Point Saloumeh, Sin el Fil, Beirut, Lebanon; 18 EHESP-School of Public Health, Paris Descartes University, 20 Avenue George Sand La Plaine, 93210 Saint Denis, France; 19 Department of Psychiatry, Chinese University of Hong Kong, Tai Po, Hong Kong, China; 20 Murcia Health Service, C/ Lorca, nº 58. -El Palmar, 30120 Murcia, Spain; 21 Colegio Mayor de Cundinamarca University, Calle 28 No. 6-02, 11001000 Bogotá, Colombia; 22 Department of Psychological Medicine, University of Otago, PO Box 56, Dunedin, 9054 New Zealand; 23 Faculty of Medicine, University of Buenos Aires, Paraguay 2155, C1121ABG CABA, Buenos Aires, Argentina; 24 Trimbos-Instituut, Netherlands Institute of Mental Health and Addiction, Da Costakade 45Postbus 725, 3500, 3521 VS Utrecht, The Netherlands; 25 Department of Psychiatry, National Taiwan University Hospital & College of Medicine, National Taiwan University, No. 7, Chung-Shan South Road, Taipei, 10002 Taiwan; 26 Lisbon Institute of Global Mental Health and Chronic Diseases Research Center (CEDOC), Nova Medical School, Universidade Nova de Lisboa, Campo Mártires da Pátria, 130, 1169-056 Lisbon, Portugal; 27 Institute for Development Research Advocacy and Applied Care (IDRAAC), Achrafieh, St. George Hospital Street, Beirut, Lebanon; 28 CIBER en Epidemiología Y Salud Pública (CIBERESP), Av. Monforte de Lemos, 3-5. Pabellón 11. Planta 0, 28029 Madrid, Spain; 29 Pompeu Fabra University (UPF), Plaça de la Mercè, 10-12, 08002 Barcelona, Spain; 30 G/F Multicentre, Tai Po Hospital, Tai Po, Hong Kong, China

**Keywords:** Mental health services, Mental health systems, Major depressive disorder, Effective coverage, Global mental health

## Abstract

**Background:**

Most individuals with major depressive disorder (MDD) receive either no care or inadequate care. The aims of this study is to investigate potential determinants of effective treatment coverage.

**Methods:**

In order to examine obstacles to providing or receiving care, the type of care received, and the quality and use of that care in a representative sample of individuals with MDD, we analyzed data from 17 WHO World Mental Health Surveys conducted in 15 countries (9 high-income and 6 low/middle-income). Of 35,012 respondents, 3341 had 12-month MDD. We explored the association of socio-economic and demographic characteristics, insurance, and severity with effective treatment coverage and its components, including type of treatment, adequacy of treatment, dose, and adherence.

**Results:**

High level of education (OR = 1.63; 1.19, 2.24), private insurance (OR = 1.62; 1.06, 2.48), and age (30–59yrs; OR = 1.58; 1.21, 2.07) predicted effective treatment coverage for depression in a multivariable logistic regression model. Exploratory bivariate models further indicate that education may follow a dose—response relation; that people with severe depression are more likely to receive any services, but less likely to receive adequate services; and that in low and middle-income countries, private insurance (the only significant predictor) increased the likelihood of receiving effective treatment coverage four times.

**Conclusions:**

In the regression models, specific social determinants predicted effective coverage for major depression. Knowing the factors that determine who does and does not receive treatment contributes to improve our understanding of unmet needs and our ability to develop targeted interventions.

**Supplementary Information:**

The online version contains supplementary material available at 10.1186/s13033-022-00539-6.

## Background

### The burden of depression

Mental disorders are the most disabling of all disorder groupings [1, 2], and result in the largest economic impact of all non-communicable disorders [3, 4]. Major depression is the leading cause of disability worldwide with an estimated 4.4 percent (approximately 322 million people) of the world’s population living with the disorder [5]. Depressed individuals are at greater risk for death from suicide, heart disease, stroke, and cancer. [6, 7] The economic costs of depression are enormous as reflected in healthcare utilization, use of social services, loss of productivity in the workplace, and loss of income and benefits for families. [8–12]

### Measuring treatment coverage for depression

Despite the availability of effective and cost-effective pharmacological and psychotherapeutic treatments for depression, [13–15] under-spending on treatment is common and the majority of individuals in need lack care [16–21]. A high priority for research is to better understand the bottlenecks or barriers that limit the number of people who receive care. Although many barriers have been well studied (e.g., stigma, mental health literacy, physical access to services), others, such as insurance, have not [22, 23]. The importance of evaluating the extent to which individuals receive effective care is heightened by a global push to achieve universal health coverage under the Sustainable Development Goals. [18–20] Several well established methodologies have been proposed across health specialties, but effective treatment coverage indicators in the area of mental health were lacking until recently. [21, 24–26]

Based on prior work by our group on minimally adequate treatment for MDD we have recently developed an “effective treatment coverage” indicator by adding adjustments for quality of care and compliance: we factor in severity-specific needs, adequacy of providers, adherence to guidelines (for psychotherapy and psychopharmacology), drug type, and adherence to the indicated dose, based on survey results from 15 countries across four continents [27, 28].

In summary, we have developed an indicator that quantifies utilization, but also includes adjustments for quality of care and user adherence to approximate outcome-based measures and allow for an estimation of potential health gains. Here we investigate how potential determinants statistically predicted the likelihood of receiving effective treatment coverage and its different components to provide a multipronged appraisal of critical obstacles to providing and receiving care.

## Methods

### Sample

The WHO World Mental Health (WHO-WMH) Surveys Initiative conducted 17 community surveys with 35,012 adult respondents in 15 countries, which include six low- or middle-income countries (LAMICs) and nine high income countries (HICs) (as per the World Bank’s classification). Samples were based on multi-stage clustered area probability household designs; they were nationally representative in 11 surveys, representative of all urbanized areas in two, and of selected regions or Metropolitan areas in the others (Table 1).

**Table 1:** WMH sample characteristics by World Bank income categories

Country^a^	Survey^b^	Sample characteristics^c^	Field dates	Age range	Sample size		Response rate^d^
					Part I	Part II	
**I**. Low and Middle-income countries							
Brazil—São Paulo	São Paulo Megacity	São Paulo metropolitan area	2005–8	18–93	5037	2942	81.3
Colombia	NSMH	All urban areas of the country (approximately 73% of the total national population)	2003	18–65	4426	2381	87.7
Colombia – Medellín	MMHHS	Medellin metropolitan area	2011–12	19–65	3261	1673	97.2
Lebanon	LEBANON	Nationally representative	2002–3	18–94	2857	1031	70.0
Mexico	M-NCS	All urban areas of the country (approximately 75% of the total national population)	2001–2	18–65	5782	2362	76.6
Nigeria	NSMHW	21 of the 36 states in the country, representing 57% of the national population. The surveys were conducted in Yoruba, Igbo, Hausa and Efik languages	2002–4	18–100	6752	2143	79.3
Romania	RMHS	Nationally representative	2005–6	18–96	2357	2357	70.9
Total					(30,472)	(14,889)	80.1
II. High-income countries							
Argentina	AMHES	Eight largest urban areas of the country (approximately 50% of the total national population)	2015	18–98	3927	2116	77.3
Belgium	ESEMeD	Nationally representative. The sample was selected from a national register of Belgium residents	2001–2	18–95	2419	1043	50.6
France	ESEMeD	Nationally representative. The sample was selected from a national list of households with listed telephone numbers	2001–2	18–97	2894	1436	45.9
Germany	ESEMeD	Nationally representative	2002–3	19–95	3555	1323	57.8
Italy	ESEMeD	Nationally representative. The sample was selected from municipality resident registries	2001–2	18–100	4712	1779	71.3
Netherlands	ESEMeD	Nationally representative. The sample was selected from municipal postal registries	2002–3	18–95	2372	1094	56.4
Portugal	NMHS	Nationally representative	2008–9	18–81	3849	2060	57.3
Spain	ESEMeD	Nationally representative	2001–2	18–98	5473	2121	78.6
Spain—Murcia	PEGASUS- Murcia	Murcia region. Regionally representative	2010–12	18–96	2621	1459	67.4
United States	NCS-R	Nationally representative	2001–3	18–99	9282	5692	70.9
Total					(41,104)	(20,123)	64.4
III. Total^e^					(71,576)	(35,012)	70.3

^a^The World Bank (2012) Data. Accessed May 12, 2012 at: http://data.worldbank.org/country. Some of the WMH countries have moved into new income categories since the surveys were conducted. The income groupings above reflect the status of each country at the time of data collection. The current income category of each country is available at the preceding URL

^b^NSMH (The Colombian National Study of Mental Health); MMHHS (Medellín Mental Health Household Study); LEBANON (Lebanese Evaluation of the Burden of Ailments and Needs of the Nation); M-NCS (The Mexico National Comorbidity Survey); NSMHW (The Nigerian Survey of Mental Health and Wellbeing); RMHS (Romania Mental Health Survey); AMHES (Argentina Mental Health Epidemiologic Survey); ESEMeD (The European Study Of The Epidemiology Of Mental Disorders); NMHS (Portugal National Mental Health Survey); PEGASUS-Murcia (Psychiatric Enquiry to General Population in Southeast Spain-Murcia);NCS-R (The US National Comorbidity Survey Replication)

^c^Most WMH surveys are based on stratified multistage clustered area probability household samples in which samples of areas equivalent to counties or municipalities in the US were selected in the first stage followed by one or more subsequent stages of geographic sampling (e.g., towns within counties, blocks within towns, households within blocks) to arrive at a sample of households, in each of which a listing of household members was created and one or two people were selected from this listing to be interviewed. No substitution was allowed when the originally sampled household resident could not be interviewed. These household samples were selected from Census area data in all countries other than France (where telephone directories were used to select households) and the Netherlands (where postal registries were used to select households). Several WMH surveys (Belgium, Germany, Italy, Spain-Murcia) used municipal, country resident or universal health-care registries to select respondents without listing households. 10 of the 17 surveys are based on nationally representative household samples

^d^The response rate is calculated as the ratio of the number of households in which an interview was completed to the number of households originally sampled, excluding from the denominator households known not to be eligible either because of being vacant at the time of initial contact or because the residents were unable to speak the designated languages of the survey. The weighted average response rate is 70.3%

^e^The following surveys, included in Thornicroft et al. [27] were excluded from this study due to lack of data on the specific drug taken and on adherence to prescribed dosage: Beijing/Shanghai, Bulgaria, Iraq, Israel, Japan, and Peru

Interviews were face-to-face and conducted in respondents’ homes by trained lay interviewers (training and quality control procedures are described elsewhere) [29]. Respondents were 18 years or older (except in Medellin, Colombia, where they were 19 +). Average response rate weighted by sample size was 70.3% following the American Association for Public Opinion Research RR1w definition [30].

Interviews were divided into two parts to reduce respondent burden. Part I assessed core mental disorders and was administered to all respondents. Part II assessed additional disorders and correlates in all Part I respondents with any disorder, plus a probability subsample of other respondents. Part II data were weighted to adjust for the under-sampling of Part I non-cases, with the resulting Part II prevalence estimates being equivalent to Part I estimates [31]. Of the 71,576 Part I and 35,012 Part II respondents, we focused our analyses on the 3341 Part II respondents with 12-month MDD. Table 2 shows the sociodemographic characteristics of our sample.

**Table 2:** Sociodemographic distribution of the sample by country-income level, among those with 12-month major depressive disorder

	All countries (n = 3341)		High income countries (n = 1991)		Low/middle income countries (n = 1350)	
	%/Mean	(SE)	%/Mean	(SE)	%/Mean	(SE)
Gender						
Male	30.4	(1.1)	31.3	(1.3)	29.1	(1.8)
Female	69.6	(1.1)	68.7	(1.3)	70.9	(1.8)
Age Group						
18–29	28.7	(1.1)	25.5	(1.4)	33.6	(1.8)
30–44	33.9	(1.0)	32.7	(1.2)	35.7	(1.8)
45–59	25.1	(0.9)	26.7	(1.2)	22.8	(1.3)
60 +	12.3	(0.7)	15.2	(1.1)	8.0	(0.9)
Marital status						
Separated, divorced, or widowed	19.8	(0.8)	20.8	(1.1)	18.4	(1.2)
Never married	26.5	(1.1)	26.1	(1.5)	27.1	(1.7)
Married or cohabitating	53.7	(1.1)	53.1	(1.5)	54.6	(1.8)
Income						
Low	31.1	(1.0)	30.5	(1.4)	32.1	(1.6)
Low-average	24.3	(0.9)	24.7	(1.2)	23.8	(1.6)
Average-high	24.0	(0.9)	26.2	(1.1)	20.8	(1.6)
High	20.5	(0.9)	18.6	(1.1)	23.4	(1.6)
Education						
Low	20.9	(0.8)	21.6	(1.1)	19.9	(1.2)
Low-average	30.1	(1.1)	33.3	(1.4)	25.3	(1.6)
Average-high	29.1	(1.0)	25.5	(1.3)	34.6	(1.7)
High	19.8	(1.0)	19.5	(1.4)	20.3	(1.4)
Insurance						
Direct private/optional insurance (yes)	17.3	(0.9)	21.5	(1.3)	11.1	(1.3)
Employment status						
Homemaker	15.6	(0.8)	9.4	(0.7)	24.8	(1.4)
Other	16.1	(0.8)	17.5	(1.1)	14.1	(1.1)
Retired	8.9	(0.6)	11.9	(0.9)	4.3	(0.8)
Student	4.7	(0.6)	4.5	(0.8)	4.9	(0.9)
Working	54.7	(1.2)	56.6	(1.6)	51.9	(1.8)
Severity						
Severe	36.8	(1.1)	36.5	(1.4)	37.1	(1.8)
Moderate	45.1	(1.1)	45.5	(1.4)	44.5	(1.7)
Mild	18.1	(0.8)	18.0	(1.1)	18.3	(1.2)
Survey year^a^						
Continuous	3.8	(0.1)	3.4	(0.2)	4.3	(0.1)

^a^Survey year is continuous, so the mean is shown instead of %

### Measures and data analysis

The survey instrument was the WHO Composite International Diagnostic Interview (CIDI) Version 3.0 [32], a fully-structured interview generating lifetime and 12-month prevalence of DSM-IV disorders, which includes protocols of translation, back-translation, adaptation, and harmonization across sites [33]. Twelve-month MDD required having a major depressive episode among respondents without a lifetime history of bipolar spectrum disorder [34]. Blinded reappraisal interviews with the Structured Clinical Interview for DSM-IV had good concordance with CIDI diagnoses [35–37]. Severity was established using trans-diagnostic criteria defined at the respondent level. Respondents with MDD were considered severe either if they had severe role impairment according to the Sheehan Disability Scale (SDS), met criteria for comorbid substance dependence with a physiological dependence syndrome, or reported a suicide attempt [38]. Respondents not considered severe were considered moderate if they reported moderate role impairment in the SDS or had substance dependence without a physiological dependence syndrome. The remaining cases were considered mild.

To build our effective treatment coverage indicator, we combined variables related to the provision of services. We classified care providers as: (1) *specialist mental health* (SMH; psychiatrist, psychologist, other mental health professional in any setting; social worker or counselor in a mental health specialized setting); or (2) *general medical* (GM; primary care doctor, other medical doctor, any other healthcare professional seen in a GM setting). Respondents provided the number of visits with each in the past 12 months and, for medical providers, clarified whether they received psychotherapy, pharmacotherapy, or both. For each psychotropic used in the past 12 months, the type, dose, and duration were recorded. Further details about the treatment variables are presented elsewhere [39].

*Contact coverage* involved any 12-month contact with a specialist or general medical provider for a mental health condition. For the pharmacotherapy measures two clinical psychiatrists with expertise in public health (DV, CSW) independently reviewed responses about medications used (which involved selecting from country specific lists including generic and brand names) and classified them. Discrepancies were reconciled by consensus. *Adequate medication control* required at least four physician visits [39]. *Medication adherence* required taking the prescribed daily dose on at least 27 out of 30 days (i.e., at least 90% of the time) [40–43]. *Adequate pharmacotherapy* required taking an antidepressant with adequate medication control and adherence (see Additional file 1: Appendix Box S1 for a list of antidepressants). A small fraction of people with MDD may avoid antidepressants due to side effects, failed trials, or other legitimate reasons, so if a non-antidepressant psychotropic was adequately controlled by a psychiatrist with adequate patient adherence, it was also considered adequate.

*Any psychotherapy* required having two or more visits to any specialty mental health provider among help seekers. *Adequate number of sessions* required at least eight sessions [39]. *Adequate psychotherapy* required at least 8 sessions from an adequate provider or still being in treatment after 2 visits. In the case of psychiatrists, for an encounter to be considered as a psychotherapeutic intervention (as opposed of medication control), visits needed to last 30 min or more.

We also defined a severity-specific variable for *effective treatment coverage,* which for mild and moderate MDD required adequate pharmacotherapy and/or adequate psychotherapy, and for severe MDD both adequate pharmacotherapy and adequate psychotherapy. These summary criteria result from a review of the National Institute for Health and Care Excellence Guidelines (NICE [44]), the Canadian Network for Mood and Anxiety Treatments guidelines (CANMAT [45, 46]), the American Psychiatric Association Practice Guideline for the Treatment of Patients With Major Depressive Disorder (APA [47]), and the WHO mhGAP Intervention Guide [48]. Table 3 shows the components of effective coverage by country and income level.

**Table 3:** Components of effective coverage among those with 12-month major depressive disorder by country income level

Coverage type		High-income countries (n = 1991)		Low/middle-income countries (n = 1350)		Significance between country income level (HICs vs LAMICs)
Among	Coverage type	%	(SE)	%	(SE)	F test
People with 12- month MDD (n = 3341)	Contact coverage	52.0	(1.5)	26.5	(1.3)	145.5*
People with contact coverage (n = 1398)	Adequate pharmacotherapy	27.6	(1.7)	22.3	(3.3)	1.7
	Any pharmacotherapy	72.9	(2.2)	57.4	(2.9)	18.0*
	Adequate psychotherapy	33.2	(1.7)	30.2	(3.4)	0.6
	Any psychotherapy	38.8	(1.7)	39.2	(3.6)	0.0
People with 12- month MDD (n = 3341)	Effective coverage	16.3	(0.9)	6.0	(0.9)	41.5*

*HICs* high-income countries; *LAMICs* low/middle-income countries; *SE* standard error; *MDD* major depressive disorder

^*^Significant at 0.05 level, two-sided test

The sample for analysis was respondents who met criteria for 12-month MDD. Differences in within-household probabilities of selection and residual discrepancies between sample and population distributions were adjusted for through weights based on census demographic-geographic variables [31]. The Taylor series linearization method [49] implemented in SUDAAN software [50] was used to estimate standard errors to adjust for weighting and geographic clustering of data.

We first ran bivariate logistic regression analyses to explore preliminary significant correlations between a specific set of potential predictors based on previous knowledge (gender, age, marital status, income, education, type of health insurance, private insurance (yes/no), employment status, severity, and survey year) and the outcome of interest, effective treatment coverage for MDD.

We then developed a multivariable logistic regression model to statistically predict effective treatment coverage including all the variables that had shown significance in the bivariate correlations. Significance was established at p < 0.05, and we report the unadjusted p values as well as values adjusted for false discovery rates (FDR) resulting from multiple testing using the Benjamini–Hochberg procedure.

Additionally, for those bivariate models that were significant in predicting “effective treatment coverage”, we conducted exploratory analyses by decomposing this indicator to identify which components may drive coverage for specific subgroups. So, we looked at determinants of contact coverage among those with 12-month MDD, and of the specific components of treatment (i.e. any pharmacotherapy, adequate pharmacotherapy, any psychotherapy, and adequate psychotherapy) among those with 12-month MDD and contact coverage. Finally, we stratified our analyses by country income level, and for people with severe MDD.

## Results

### Main analysis

Significant predictors of effective treatment coverage for persons with MDD.

In our initial bivariate models, the following variables were associated with effective treatment coverage: age, income, education, type of insurance, private insurance, and severity. After adjusting for the FDR, age, education, type of insurance and private insurance remained significant, while income and severity were not statistically significant (p = 0.055 and 0.073 respectively) (see Table 4).

**Table 4:** Bivariate predictors of effective coverage and its components among those with 12-Month major depressive disorder, in all countries (n = 3341)

	Among those with 12-month MDD (n=3,341), received contact coverage^a^				Among those with contact coverage (n=1398)												Among those with 12-month MDD (n=3341), received effective coverage			
					Received any pharmacotherapy			Received adequate pharmacotherapy			Received any psychotherapy			Received adequate psychotherapy						
	OR	(95% CI)		F test	OR	(95% CI)	F test	OR	(95% CI)	F test	OR	(95% CI)	F test	OR	(95% CI)	F test	OR	(95% CI)	F test	FDR†
Age																				
18-29	*0.8 *	(0.6-1.1)		10.4*	0.4*	(0.2-0.7)	9.9*	0.8	(0.5-1.4)	1.7	2.9*	(1.7-5.0)	6.1*	2.6*	(1.5-4.3)	5.1*	1.1	(0.6-1.8)	3.6*	0.041
30-44	*1.1 *	(0.8-1.5)			0.8	(0.4,1.3)		1.2	(0.8-2.0)		2.9*	(1.7-4.8)		2.8*	(1.6-4.7)		1.6*	(1.0-2.6)		
45-59	1.5*	(1.1-2.1)			1.2	(0.7-2.0)		1.3	(0.9-2.0)		2.0*	(1.2-3.4)		1.9*			1.6*	(1.0-2.6)		
60+ (Ref)	*REF*				REF			REF			REF			REF			REF			
Income																				
Low	0.7*	(0.5-0.9)		3.0*	0.9	(0.6-1.4)	0.7	0.8	(0.5–1.3)	0*.7*	0.6*	(0.4–0.9)	2.8*	0.6	(0.4–1.0)	2*.3*	0.6*	(0.4–0.8)	3.3*	0.055
Low-average	0.7*	(0.5–1.0)			0.7	(0.4–1.2)		0.9	(0.6,–.5)		0.8	(0.5–1.3)		0.8	*(*0.5–1.3)		0.8	(0.5–1.1)		
Average-high	0.7*	(0.6–0.9)			0.8	(0.5–1.3)		0.7	(0.5–1.1)		0.6*	(0.4–0.9)		0.6*	(0.4–1.0)		0.6*	(0.4–0.9)		
High (Ref)	REF				REF			REF			EF			REF			REF			
Level of education																				
Low	0.8	(0.6–1.1)		3.0*	1.2	(0.7–1.9)	0.2	0.6*	(0.4–1.0)	2.0	0.4*	*(*0.3–0.7)	7.3*	0.5*	(0.3–0.7)	5.6*	0.4*	(0.3–0.6)	7.0*	0.001
Low-average	0.7*	(0.5–0.9)			1.0	(0.6–1.6)		0.8	(0.5–1.1)		0.6*	(0.4–0.9)		0.5*	(0.3–0.8)		0.6*	(0.4–0.8)		
Average-high	0.7*	(0.5–0.9)			1.2	(0.7–1.8)		1.0	(0.7–1.6)		0.9	(0.6–1.4)		0.9	(0.6–1.4)		0.8	(0.6–1.1)		
High (Ref)	REF				REF			REF			REF			REF			REF			
Type of insurance																				
None (Ref)	REF				REF			REF			REF			REF			REF			
Direct private/optional insurance	2.2*	(1.4–3.2)		6.8	1.1	(0.6–2.1)	0.1	0.9	(0.4–1.8)	0.2	1.4	(0.7–2.6)	2.8	1.7	(0.8–3.4)	3.7*	2.4*	(1.2–5.0)	4.3*	0.042
Any other types of insurance	1.3	(1.0–1.8)			1.1	(0.7–1.9)		0.8	(0.4–1.5)		0.8	(0.4–1.5)		0.9	(0.5–1.8)		1.4	(0.7–2.5)		
Insurance																				
Direct private/optional insurance (yes)	1.7*	(1.2–2.4)	9.8*		1.0	(0.7–1.5)	0.0	1.0	(0.6–1.7)	0.1	1.6*	(1.1–2.4)	5.3*	1.8*	(1.2–2.7)	7.4*	1.8*	(1.2–2.8)	7.8*	0.022
Severity																				
Severe (Ref.)	REF				REF			REF			REF			REF			REF			
Moderate	0.5*	(0.4–0.6)	35.1*		0.7*	(0.5–0.9)	8.2*	0.6*	(0.4–0.9)	5.8*	0.7	(0.5–1.0)	4.8*	0.7	(0.5–1.0)	5.2*	1.4*	(1.0–1.9)	3.4*	0.073
Mild	0.4*	(0.3–0.5)			0.4*	(0.2–0.6)		0.5*	(0.3–0.8)		0.5*	(0.3–0.8)		0.5*	(0.3–0.8)		0.9	(0.6–1.4)		

*MDD* major depressive disorder; *OR* odds ratio; *CI* confidence interval

^*^Significant at the 0.05 level, two-sided test

^a^Models are bivariate with each demographic predictor in separate models, controlling for country dummies. The following variables were non-significant: gender, marital status, employment status and survey year

^†^FDR: False discovery rate adjustment for multiple testing implementing the Benjamini-Hockberg method

Our multivariate model includes all the variables that showed significance in the bivariate logistic regression analyses. In this exploratory analysis, we simplified these variables by creating dummies capturing the values that were significantly associated with increased odds of receiving effective treatment coverage: middle age or not, high income or not, average-high to high education or not, direct private insurance or not. We retained MDD severity as an ordinal variable. Table 5 shows the results: only middle age (OR = 1.6; *p* =  < 001), high or average high education (OR = 1.6; *p* = 0.002), and direct private insurance (OR = 1.6; *p* = 0.025) retain significance, while income and severity lose significance.

**Table 5:** Multivariate model of effective coverage among those with 12-month major depressive disorder, in all countries (n = 3341)

	Among those with 12-month MDD (n = 3341), received effective coverage^a^			
	OR	(95% CI)	F test	FDR†
Age				
Middle Age (30–59) Y/N	1.6*	(1.2–2.1)	11.0*	0.004
Income				
High Income Y/N	1.3	(0.9–1.8)	1.6	0.208
Level of education				
Average-high to high education, Y/N	1.6*	(1.2–2.2)	9.2*	0.006
Type of insurance				
Direct private/optional insurance, Y/N	1.6*	(1.1–2.5)	5.0*	0.042
Severity				
REF: Severe				
Moderate	1.3	(1.0–1.8)	2.3	0.127
Mild	0.9	(0.6–1.4)		
Global F test for multivariate model			5.8*	

*MDD* major depressive disorder; *OR* odds ratio; *CI* confidence interval

^*^Significant at the .05 level, two-sided test

^a^Model is a multivariate model with all rows in the same model, controlling for country dummies

^†^FDR: False discovery rate adjustment for multiple testing implementing the Benjamini-Hockberg method

### Exploratory analyses

For the variables mentioned above (which were significantly associated with effective coverage in the bivariate analysis), we conducted additional exploratory analyses of the different components of effective coverage. Five findings of potential interest stand out.

First, persons between 30 and 59 years with MDD were more likely than other age groups to get effective treatment coverage for MDD. Among help-seekers, the 18–29 group is significantly less likely to get any pharmacotherapy, followed by the 30–44, the 60 + , and again with the 45–59 being the most likely to receive it. The 60 + help-seekers are the least likely to get any psychotherapy and to get adequate psychotherapy, with other age-groups being two to three times more likely to receive either. The 45–59 group might be the most likely to receive effective treatment coverage because they are more likely to contact services (see Table 4 for details).

Second, with respect to individual-level income, persons with high income are more likely to get any contact coverage. People with the highest individual income are also significantly more likely to get effective treatment coverage than any other subgroup (see Table 4 for details).

Third, persons with highest levels of education are most likely to get effective treatment coverage, with a dose–response relationship. Interestingly, with respect to contact coverage, people with the lowest level of education do not significantly differ from those with higher education, and the inequality seems to stem from the inadequacy of the pharmacotherapy and psychotherapy, which results in the fact that those with low level of education are less than half as likely to get effective treatment coverage (see Table 4 for details).

Fourth, persons with direct private insurance are more than twice as likely to get effective treatment coverage as those with no insurance (i.e., who would need to pay out of pocket). This inequality seems to be driven by the increased likelihood of getting contact coverage, of getting any psychotherapy and adequate psychotherapy for those with private insurance (see Table 4 for details).

Fifth, persons with moderate disorders are the most likely to receive effective treatment coverage. The reason is that even though people with severe depression are more likely to have any contact coverage, any/adequate psychotherapy, or any/adequate pharmacotherapy, they are less likely to receive the adequate combination of pharmacotherapy and psychotherapy that they need. Whereas people with moderate depression are less likely to get any services, but more likely for these services to meet the more basic package they require. Persons with mild disorders receive significantly less of any and all service components (see Table 4 for details).

### Country-income level and severity

In HICs, both age and education were significant determinants of effective treatment coverage. (see Additional file 1: Appendix Table S1 for details). Thirty to 59 year-old and higher educated people with MDD receive are more likely to receive effective treatment coverage. In LAMICs, the only significant predictor of effective treatment coverage was having direct private insurance: patients with direct private insurance were four times more likely to receive effective treatment coverage than all others (*p* = 0.008; see Additional file 1: Appendix Table S2 for details).

To better understand the exact reasons why severely affected persons with MDD did not obtain treatment that meets the criteria for effective treatment coverage, we additionally studied severe MDD cases in all countries and in HICs and LAMICs separately. For severely affected people across countries, a high personal income doubled the likelihood of receiving any contact coverage (*p* < 0.01). For people receiving any services, having direct private insurance doubled the likelihood of receiving adequate psychotherapy (*p* = 0.02), and being female doubled the likelihood of receiving any psychopharmacology (*p* = 0.009). Finally, people aged 45 to 59 were the most likely to receive contact coverage (*p* = 0.018). See Additional file 1: Appendix Table S3 for details.

In HICs, severely affected people aged 45 to 59 were also more likely to have contact coverage (*p* < 0.01) and receive any pharmacotherapy (*p* = 0.017). People with private insurance were 5.6 times more likely to receive any pharmacotherapy compared to people without insurance (*p* = 0.042). See Additional file 1: Appendix Table S4 for details.

Focusing on the coverage for severely affected people in LAMICs our findings indicate that men (OR = 1.7; *p* = 0.018), people with high income (reference group, more than twice as likely than all other groups; *p* = 0.02), high education (reference group; *p* = 0.019), and direct private insurance were more likely to have contact coverage (OR = 3.6; *p* = 0.003). Further, people with direct private insurance were nearly four times more likely to get any and adequate psychotherapy (*p* = 0.045 and 0.034 respectively). Finally, married people were significantly less likely to receive any psychotherapy (*p* = 0.023), adequate psychotherapy (*p* = 0.048), and adequate pharmacotherapy (*p* = 0.032). See Additional file 1: Appendix Table S5 for details.

## Discussion

Though our initial bivariate models indicate that age, income, education, insurance, and severity may be associated with effective treatment coverage for depression, after inclusion in a multivariable model and adjustment for multiple testing, only some of these variables retain significance: being 30 to 59 years old, having higher education levels, and having direct private insurance significantly contribute to increased likelihood of receiving effective treatment coverage.

Our exploratory analyses suggest that in LAMICs the only significant association with effective treatment coverage may be having private insurance. Also, for the most severely affected people in LAMICs, being a man, having high income, high education, and direct private insurance are all significantly associated with receiving contact coverage, a precondition of effective treatment coverage.

Our study adds critical information by integrating subject and demographic variables, severity of depression, type of insurance, and adequacy of care, all leading to an increased understanding of effective treatment coverage and its determinants. Our findings also raise relevant policy questions. First, the fact that education level is a determinant of effective treatment coverage offers potentially interesting areas of intervention. A known barrier to care for mental services is mental health literacy [51]. This refers to knowledge about dysfunction, resources, and the means through which they are accessed. Also, lower levels of education make it more difficult to identify sadness, diminished pleasure, loss of energy, feelings of worthlessness or guilt as medical conditions that may need treatment. Or, even if identified as such, the ability to activate the organizational levers required to receive such care may depend on a nuanced understanding of how the health care system works, and of users’ rights to healthcare in different settings. Each of these facets relate to mental health literacy, providing a parsimonious interpretation of the effect. Also, the findings convey the increased need to more assertively and responsively provide services for those at lower educational levels and limited mental health literacy. Promising work is well underway toward this end, as for example, with the use of digital mental health services [52, 53].

Second, it is not clear why private insurance was the only form of financial protection significantly associated with effective treatment coverage. “Any other type of insurance”, which included social security and publicly funded healthcare, was not significantly different from “no insurance” in our bivariate analyses. In HICs, we found that both “direct private” and “any other insurance” were significantly different from “no insurance’ when it comes to the provision of contact coverage, but that difference is lost as we adjust for the quality of those services. In LAMICs, the odds of receiving effective treatment coverage were 3.8 with “direct private insurance” vs all other. One hypothesis would be that other forms of insurance in most of these LAMICs are insufficiently developed to significantly increase even contact coverage, let alone effective treatment coverage. Another hypothesis would be that the quality of mental health care was only meaningfully better with private insurance, particularly in LAMICs. In addition, it is possible that private insurance covaries with education and income and our study showed these factors very much relate to effective treatment coverage. Ultimately, in the multivariable model pooling all countries direct private insurance significantly increased the odds of effective treatment coverage.

There are important limitations to note. First, service utilization and adherence data relied on self-reports that may be biased. We focused on 12-month treatment rather than longer recall periods to minimize recall bias. More stringent methods (e.g., blood samples, pill counts) are impractical for population-level investigations, making surveys acceptable to assess adherence. 80% and 90% have previously been used as compliance thresholds [40–42], so we used the most stringent one (taking the indicated dose at least 90% of the time) to compensate for potential bias. Additionally, given that our surveys span 15 years (2001 to 2015) and all country income levels, we have not included computer-, peer-, or community provider-delivered interventions due to their inconsistency across time and countries.

Also, many other critical variables might well influence the variables we investigated. It was not possible through our data to establish the relative importance of the many other health system, socioeconomic, and environmental variables that may determine utilization patterns.

Finally, there are limitations to both theory-driven multivariable models and those that result from retaining significant bivariate correlations. We chose a model building strategy that combines both approaches in a purposeful manner, and clearly describe each step. The rationale for conducting preliminary bivariate analyses on a set of potentially relevant variables was twofold. First, in our experience, some of these variables are highly correlated, and including them all in a purely theory driven multivariable model may show spurious significant correlations. Second, these bivariate associations are also relevant to then explore the associations with the components of our composite variable of interest. Further, given that we explore a limited number of variables, the likelihood of random significance is minimal.

## Conclusions

In summary, our findings suggest that improving financial protection may expand effective treatment coverage going beyond the direct impact of individual income. However, the findings also show that state funded health care and social security in the real world seem to expand contact coverage but are not significantly different from no insurance when an adjustment is made for the quality of services rendered. In addition, the significant impact of having a higher education calls into question the accessibility of services and may justify population-level interventions to counter stigma, decrease barriers, and increase acceptability of services. Finally, addressing entrenched sources of inequality, such as gender, income and education may be of particular importance for severely affected patients in LAMICs.

## Supplementary Information

**Additional file 1: Table S1.** Bivariate predictors of effective coverage and its components among those with 12-month major depressive disorder, in high income countries (n=1991)^1^. **Table S2.** Bivariate predictors of effective coverage and its components among those with 12-month major depressive disorder, in low/middle-income countries (n=1350)^1^. **Table S3.** Bivariate predictors of effective coverage and its components among those with 12-month major depressive disorder, in all countries, among severe cases (n=1244)^1^. **Table S4.** Bivariate predictors of effective coverage and its components among those with 12-month major depressive disorder, in high income countries, among severe cases (n=730)^1^. **Table S5.** Bivariate predictors of effective coverage and its components among those with 12-month major depressive disorder, in low and middle countries, among severe cases (n=514)^1^. **Box S1.** Antidepressants and classes

## Data Availability

Access to the cross-national World Mental Health (WMH) data is governed by the organizations funding and responsible for survey data collection in each country. These organizations made data available to the WMH consortium through restricted data sharing agreements that do not allow us to release the data to third parties. The exception is that the U.S. data are available for secondary analysis via the Inter-University Consortium for Political and Social Research (ICPSR), http://www.icpsr.umich.edu/icpsrweb/ICPSR/series/00527.

## Abbreviations

MDD: Major depressive disorder
WHO-WMH: World Health Organization World Mental Health
OR: Odds ratio
LAMICs: Low and middle income countries
HICs: High income countries
RR1w: Response rate 1, weighted
DSM-IV: Diagnostic and Statistical Manual of Mental Disorders, fourth edition
CIDI: Composite international diagnostic interview
SDS: Sheehan disability scale
SMH: Specialist mental health services
GM: General medical services
NICE: National Institute for Health and Care Excellence Guidelines
CANMAT: Canadian Network for Mood and Anxiety Treatments guidelines
APA: American Psychiatric Association
mhGAP: Mental health gap guide
FDR: False discovery rate
