# Supplementary material for: Determinants of effective treatment coverage for major depressive disorder in the WHO World Mental Health Surveys
Source: Int J Ment Health Syst. 2022 Jun 23;16:29. doi: 10.1186/s13033-022-00539-6 (PMC9219212; doi:10.1186/s13033-022-00539-6)
Supplement: Supplementary file 1 — Additional file 1: Table S1. Bivariate predictors of effective coverage and its components among those with 12-month major depressive disorder, in high income countries (n=1991)1. Table S2. Bivariate predictors of effective coverage and its components among those with 12-month major depressive disorder, in low/middle-income countries (n=1350)1. Table S3. Bivariate predictors of effective coverage and its components among those with 12-month major depressive disorder, in all countries, among severe cases (n=1244)1. Table S4. Bivariate predictors of effective coverage and its components among those with 12-month major depressive disorder, in high income countries, among severe cases (n=730)1. Table S5. Bivariate predictors of effective coverage and its components among those with 12-month major depressive disorder, in low and middle countries, among severe cases (n=514)1. Box S1. Antidepressants and classes [file 13033_2022_539_MOESM1_ESM.docx]

| **Appendix Table S1. Bivariate predictors of effective coverage and its components among those with 12-month major depressive disorder, in high income countries (n=1,991)^1^** | | | | | | | | | | | | | | | | | | |
| --- | --- | --- | --- | --- | --- | --- | --- | --- | --- | --- | --- | --- | --- | --- | --- | --- | --- | --- |
|  | | | | | | | | | | | | | | | | | | |
|  | **Among those with 12-month MDD (n=1,991), received contact coverage** | | | **Among those with 12-month MDD and contact coverage (n=1,043), received any pharmacotherapy** | | | **Among those with 12-month MDD and contact coverage (n=1,043), received adequate pharmacotherapy** | | | **Among those with 12-month MDD and contact coverage (n=1,043), received any psychotherapy** | | | **Among those with 12-month MDD and contact coverage (n=1,043), received adequate psychotherapy** | | | **Among those with 12-month MDD (n=1,043), received effective coverage** | | |
|  | **OR** | **(95% CI)** | **F test** | **OR** | **(95% CI)** | **F test** | **OR** | **(95% CI)** | **F test** | **OR** | **(95% CI)** | **F test** | **OR** | **(95% CI)** | **F test** | **OR** | **(95% CI)** | **F test** |
| Gender |  |  |  |  |  |  |  |  |  |  |  |  |  |  |  |  |  |  |
| Male | 0.8* | (0.6-1.0) | 5.1* | 0.8 | (0.5-1.1) | 1.7 | 1.0 | (0.6-1.4) | 0.1 | 1.1 | (0.8-1.6) | 0.4 | 1.1 | (0.8-1.6) | 0.2 | 0.8 | (0.6-1.1) | 2.4 |
| Female (Ref) | REF |  |  | REF |  |  | REF |  |  | REF |  |  | REF |  |  | REF |  |  |
| Age |  |  |  |  |  |  |  |  |  |  |  |  |  |  |  |  |  |  |
| 18-29 | 0.9 | (0.6-1.3) | 7.8* | 0.3* | (0.2-0.6) | 8.3* | 0.7 | (0.4-1.2) | 1.5 | 2.9* | (1.6-5.3) | 5.1* | 2.3* | (1.3-4.2) | 3.6* | 1.0 | (0.6-1.8) | 2.6* |
| 30-44 | 1.2 | (0.9-1.7) |  | 0.5* | (0.3-1.0) |  | 1.1 | (0.6-1.8) |  | 2.9* | (1.7-5.2) |  | 2.6* | (1.4-4.7) |  | 1.6 | (1.0-2.6) |  |
| 45-59 | 1.6* | (1.2-2.3) |  | 0.9 | (0.5-1.7) |  | 1.1 | (0.7-1.8) |  | 2.0* | (1.1-3.4) |  | 1.7 | (1.0-3.0) |  | 1.5 | (0.9-2.4) |  |
| 60+ (Ref) | REF |  |  | REF |  |  | REF |  |  | REF |  |  | REF |  |  | REF |  |  |
| Marital status |  |  |  |  |  |  |  |  |  |  |  |  |  |  |  |  |  |  |
| Separated, divorced, or widowed | 1.5* | (1.1-1.9) | 4.6* | 1.1 | (0.7-1.7) | 3.3* | 0.7 | (0.4-1.0) | 3.1* | 0.8 | (0.5-1.0) | 3.3* | 0.8 | (0.6-1.2) | 1.7 | 0.8 | (0.6-1.1) | 0.9 |
| Never married | 0.9 | (0.7-1.2) |  | 0.6* | (0.4-1.0) |  | 0.6 | (0.4-1.0) |  | 1.2 | (0.8-1.9) |  | 1.2 | (0.8-1.8) |  | 0.9 | (0.6-1.3) |  |
| Married or cohabiting (Ref) | REF |  |  | REF |  |  | REF |  |  | REF |  |  | REF |  |  | REF |  |  |
| Income |  |  |  |  |  |  |  |  |  |  |  |  |  |  |  |  |  |  |
| Low | 0.9 | (0.7-1.3) | 0.1 | 0.6 | (0.4-1.0) | 1.2 | 1.0 | (0.6-1.6) | 0.4 | 0.6* | (0.4-1.0) | 2.6 | 0.6 | (0.4-1.0) | 3.2* | 0.6* | (0.4-1.0) | 2.5 |
| Low-Average | 0.9 | (0.7-1.3) |  | 0.6 | (0.4-1.2) |  | 1.1 | (0.6-1.9) |  | 1.0 | (0.6-1.7) |  | 1.1 | (0.6-1.8) |  | 0.9 | (0.6-1.4) |  |
| Average-High | 1.0 | (0.7-1.3) |  | 0.7 | (0.4-1.2) |  | 0.9 | (0.5-1.4) |  | 0.7 | (0.4-1.1) |  | 0.7 | (0.4-1.1) |  | 0.7 | (0.4-1.1) |  |
| High (Ref) | REF |  |  | REF |  |  | REF |  |  | REF |  |  | REF |  |  | REF |  |  |
| Level of education |  |  |  |  |  |  |  |  |  |  |  |  |  |  |  |  |  |  |
| Low | 1.0 | (0.7-1.3) | 1.5 | 0.8 | (0.5-1.6) | 0.5 | 0.7 | (0.5-1.2) | 1.5 | 0.4* | (0.3-0.7) | 6.2* | 0.4* | (0.3-0.7) | 5.1* | 0.4* | (0.3-0.6) | 5.6* |
| Low-Average | 0.8 | (0.6-1.1) |  | 0.7 | (0.4-1.2) |  | 0.7 | (0.4-1.0) |  | 0.6* | (0.4-0.9) |  | 0.5* | (0.3-0.8) |  | 0.5* | (0.3-0.8) |  |
| Average-High | 0.7 | (0.5-1.0) |  | 0.8 | (0.4-1.5) |  | 1.0 | (0.6-1.7) |  | 0.9 | (0.6-1.4) |  | 0.9 | (0.6-1.4) |  | 0.8 | (0.5-1.1) |  |
| High (Ref) | REF |  |  | REF |  |  | REF |  |  | REF |  |  | REF |  |  | REF |  |  |
| Type of insurance |  |  |  |  |  |  |  |  |  |  |  |  |  |  |  |  |  |  |
| None (Ref) | REF |  |  | REF |  |  | REF |  |  | REF |  |  | REF |  |  | REF |  |  |
| Direct Private/Optional Insurance | 1.8* | (1.1-2.8) | 3.7* | 2.0* | (1.0-4.1) | 2.4 | 0.8 | (0.4-1.9) | 0.1 | 1.0 | (0.5-2.2) | 0.3 | 1.2 | (0.6-2.6) | 0.4 | 2.2 | (1.0-5.1) | 2.1 |
| Any other types of insurance | 1.6* | (1.1-2.2) |  | 1.8 | (1.0-3.4) |  | 2.0 | (0.5-2.0) |  | 0.9 | (0.4-1.8) |  | 1.0 | (0.4-2.0) |  | 1.6 | (0.7-3.5) |  |
| Insurance |  |  |  |  |  |  |  |  |  |  |  |  |  |  |  |  |  |  |
| Direct Private/Optional Insurance (Yes) | 1.2 | (0.8-1.7) | 0.8 | 1.2 | (0.7-2.0) | 0.5 | 0.9 | (0.5-1.5) | 0.3 | 1.2 | (0.7-1.9) | 0.4 | 1.2 | (0.8-2.0) | 0.8 | 1.4 | (1.0-2.2) | 3.2 |
| **Appendix Table S1 continued. Bivariate predictors of effective coverage and its components among those with 12-month major depressive disorder, in high income countries (n=1,991)^1^** | | | | | | | | | | | | | | | | | | |
|  | | | | | | | | | | | | | | | | | | |
|  | **Among those with 12-month MDD (n=1,991), received contact coverage** | | | **Among those with 12-month MDD and contact coverage (n=1,043), received any pharmacotherapy** | | | **Among those with 12-month MDD and contact coverage (n=1,043), received adequate pharmacotherapy** | | | **Among those with 12-month MDD and contact coverage (n=1,043), received any psychotherapy** | | | **Among those with 12-month MDD and contact coverage (n=1,043), received adequate psychotherapy** | | | **Among those with 12-month MDD (n=1,043), received effective coverage** | | |
|  | **OR** | **(95% CI)** | **F test** | **OR** | **(95% CI)** | **F test** | **OR** | **(95% CI)** | **F test** | **OR** | **(95% CI)** | **F test** | **OR** | **(95% CI)** | **F test** | **OR** | **(95% CI)** | **F test** |
| Employment status |  |  |  |  |  |  |  |  |  |  |  |  |  |  |  |  |  |  |
| Homemaker | 0.7 | (0.5-1.0) | 4.4* | 1.9* | (1.0-3.6) | 2.0 | 1.8 | (1.0-3.2) | 2.2 | 1.1 | (0.6-2.0) | 2.6* | 1.1 | (0.6-2.0) | 1.9 | 0.9 | (0.6-1.5) | 1.0 |
| Other | 1.6* | (1.2-2.3) |  | 1.1 | (0.7-1.7) |  | 1.7* | (1.1-2.6) |  | 1.3 | (0.9-1.9) |  | 1.2 | (0.8-1.9) |  | 1.3 | (0.9-1.9) |  |
| Retired | 1.0 | (0.7-1.4) |  | 1.9* | (1.0-3.6) |  | 1.3 | (0.8-2.2) |  | 0.7 | (0.4-1.1) |  | 0.8 | (0.4-1.2) |  | 0.9 | (0.6-1.4) |  |
| Student | 0.7 | (0.3-1.3) |  | 0.7 | (0.2-2.2) |  | 1.0 | (0.4-3.0) |  | 2.7* | (1.2-6.1) |  | 2.6* | (1.1-6.2) |  | 1.3 | (0.6-3.0) |  |
| Working (Ref) | REF |  |  | REF |  |  | REF |  |  | REF |  |  | REF |  |  | REF |  |  |
| Severity |  |  |  |  |  |  |  |  |  |  |  |  |  |  |  |  |  |  |
| Severe (Ref) | REF |  |  | REF |  |  | REF |  |  | REF |  |  | REF |  |  | REF |  |  |
| Moderate | 0.5* | (0.4-0.7) | 21.7* | 0.6* | (0.4-0.9) | 6.0* | 0.5* | (0.4-0.8) | 6.4* | 0.7 | (0.5-1.1) | 1.6 | 0.7 | (0.5-1.0) | 2.0 | 1.5* | (1.0-2.1) | 2.4 |
| Mild | 0.4* | (0.2-0.5) |  | 0.4* | (0.2-0.7) |  | 0.4* | (0.3-0.9) |  | 0.7 | (0.4-1.2) |  | 0.6 | (0.4-1.1) |  | 1.1 | (0.7-1.7) |  |
| Survey year |  |  |  |  |  |  |  |  |  |  |  |  |  |  |  |  |  |  |
| Continuous | 1.3 | (1.0-1.8) | 3.0 | 1.0 | (0.7-1.5) | 0.0 | 1.5* | (1.1-2.0) | 5.8* | 1.3 | (0.9-1.8) | 2.2 | 1.2 | (0.8-1.7) | 0.9 | 1.4 | (0.9-2.0) | 2.6 |
|  |  |  |  |  |  |  |  |  |  |  |  |  |  |  |  |  |  |  |

Abbreviations. MDD, major depressive disorder; OR, odds ratio; CI, confidence interval.

*Significant at the .05 level, two-sided test.

^1^Models are bivariate models with each demographic predictors in separate models, controlling for country dummies.

| **Appendix Table S2. Bivariate predictors of effective coverage and its components among those with 12-month major depressive disorder, in low/middle-income countries (n=1350)^1^** | | | | | | | | | | | | | | | | | | |
| --- | --- | --- | --- | --- | --- | --- | --- | --- | --- | --- | --- | --- | --- | --- | --- | --- | --- | --- |
|  |  | | |  | | |  | | |  | | |  | | |  | | |
|  | **Among those with 12-month MDD (n=1,350), received contact coverage** | | | **Among those with 12-month MDD and contact coverage (n=355), received any pharmachotherapy** | | | **Among those with 12-month MDD and contact coverage (n=355), received adequate pharmacotherapy** | | | **Among those with 12-month MDD and contact coverage (n=355), received any psychotherapy** | | | **Among those with 12-month MDD and contact coverage (n=355), received adequate psychotherapy** | | | **Among those with 12-month MDD (n=1,350), received effective coverage** | | |
|  | **OR** | **(95% CI)** | **F test** | **OR** | **(95% CI)** | **F test** | **OR** | **(95% CI)** | **F test** | **OR** | **(95% CI)** | **F test** | **OR** | **(95% CI)** | **F test** | **OR** | **(95% CI)** | F test |
| Gender |  |  |  |  |  |  |  |  |  |  |  |  |  |  |  |  |  |  |
| Male | 1.2 | (0.8-1.6) | 1.0 | 0.7 | (0.3-1.4) | 1.1 | 1.2 | (0.5-2.8) | 0.2 | 0.9 | (0.4-1.8) | 0.1 | 1.0 | (0.5-2.2) | 0.0 | 1.0 | (0.4-2.2) | 0.0 |
| Female (Ref) | REF |  |  | REF |  |  | REF |  |  | REF |  |  | REF |  |  | REF |  |  |
| Age |  |  |  |  |  |  |  |  |  |  |  |  |  |  |  |  |  |  |
| Continuous | 1.0* | (1.0-1.0) | 9.1* | 1.0 | (1.0-1.0) | 2.3 | 1.0 | (1.0-1.0) | 0.1 | 1.0 | (1.0-1.0) | 2.8 | 1.0 | (1.0-1.0) | 2.9 | 1.0 | (1.0-1.0) | 1.2 |
| Marital status |  |  |  |  |  |  |  |  |  |  |  |  |  |  |  |  |  |  |
| Separated, divorced, or widowed | 1.0 | (0.7-1.4) | 0.7 | 1.1 | (0.5-2.2) | 0.5 | 1.0 | (0.5-2.2) | 0.3 | 1.2 | (0.6-2.4) | 3.1* | 1.1 | (0.5-2.4) | 1.6 | 1.0 | (0.4-2.0) | 0.1 |
| Never married | 0.8 | (0.5-1.2) |  | 0.6 | (0.3-1.6) |  | 1.4 | (0.6-3.2) |  | 2.8* | (1.2-6.3) |  | 2.3 | (0.9-5.9) |  | 1.1 | (0.6-2.1) |  |
| Married or cohabiting (Ref) | REF |  |  | REF |  |  | REF |  |  | REF |  |  | REF |  |  | REF |  |  |
| Income |  |  |  |  |  |  |  |  |  |  |  |  |  |  |  |  |  |  |
| Low | 0.4* | (0.3-0.7) | 7.7* | 2.2 | (1.0-5.0) | 2.9* | 0.6 | (0.2-1.6) | 0.7 | 0.6 | (0.2-1.5) | 1.5 | 0.8 | (0.3-2.1) | 2.5 | 0.4 | (0.1-1.0) | 1.7 |
| Low-Average | 0.5* | (0.3-0.8) |  | 0.7 | (0.3-1.7) |  | 0.6 | (0.2-1.6) |  | 0.5 | (0.2-1.3) |  | 0.4 | (0.2-1.0) |  | 0.4 | (0.1-1.0) |  |
| Average-High | 0.4* | (0.3-0.6) |  | 1.0 | (0.4-2.2) |  | 0.5 | (0.2-1.4) |  | 0.5 | (0.2-1.0) |  | 0.5 | (0.2-1.2) |  | 0.4 | (0.2-1.1) |  |
| High (Ref) | REF |  |  | REF |  |  | REF |  |  | REF |  |  | REF |  |  | REF |  |  |
| Level of education |  |  |  |  |  |  |  |  |  |  |  |  |  |  |  |  |  |  |
| Low | 0.6 | (0.4-1.0) | 3.2* | 2.0 | (0.9-4.7) | 2.0 | 0.2 | (0.1-1.0) | 2.0 | 0.5 | (0.2-1.2) | 1.3 | 0.5 | (0.2-1.5) | 0.8 | 0.3 | (0.1-1.0) | 1.6 |
| Low- Average | 0.5* | (0.3-0.8) |  | 2.5* | (1.0-6.0) |  | 1.1 | (0.4-2.9) |  | 0.5 | (0.2-1.2) | 1.3 | 0.5 | (0.2-1.5) | 0.8 | 0.3 | (0.1-1.0) | 1.6 |
| Average- High | 0.7 | (0.4-1.5) |  | 2.2* | (1.1-4.3) |  | 1.0 | (0.4-2.4) |  | 1.0 | (0.4-2.4) |  | 1.0 | (0.5-2.4) |  | 0.9 | (0.4-1.9) |  |
| High (Ref) | REF |  |  | REF |  |  | REF |  |  | REF |  |  | REF |  |  | REF |  |  |
| Insurance |  |  |  |  |  |  |  |  |  |  |  |  |  |  |  |  |  |  |
| Direct Private/Optional Insurance (Yes) | 3.4* | (1.9-6.0) | 16.9* | 0.8 | (0.4-1.6) | 0.4 | 1.6 | (0.6-4.1) | 1.0 | 3.0* | (1.4-6.3) | 8.4* | 3.6* | (1.7-7.7) | 11.3* | 3.8* | (1.4-10.2) | 7.0* |
| Employment status |  |  |  |  |  |  |  |  |  |  |  |  |  |  |  |  |  |  |
| Working (Yes) | 0.7* | (0.5-1.0) | 5.2* | 0.7 | (0.4-1.0) | 3.3 | 0.6 | (0.3-1.3) | 1.7 | 1.0 | (0.6-1.7) | 0.0 | 0.9 | (0.5-1.7) | 0.1 | 0.6 | (0.3-1.1) | 2.9 |
| Severity |  |  |  |  |  |  |  |  |  |  |  |  |  |  |  |  |  |  |
| Severe (Ref) | REF |  |  | REF |  |  | REF |  |  | REF |  |  | REF |  |  | REF |  |  |
| Moderate | 0.4* | (0.3-0.6) | 15.6* | 0.8 | (0.4-1.4) | 2.5 | 1.1 | (0.4-2.6) | 0.8 | 0.8 | (0.4-1.4) | 7.8* | 0.9 | (0.5-1.7) | 5.4* | 1.3 | (0.8-2.2) | 1.4 |
| Mild | 0.4* | (0.2-0.6) |  | 0.4* | (0.2-0.9) |  | 0.6 | (0.2-1.4) |  | 0.2* | (0.1-0.5) |  | 0.2* | (0.0-0.5) |  | 0.5 | (0.2-1.4) |  |
| Survey year |  |  |  |  |  |  |  |  |  |  |  |  |  |  |  |  |  |  |
| Continuous | 0.8 | (0.4-1.3) | 1.0 | 0.8 | (0.3-2.3) | 0.1 | 1.3 | (0.2-7.4) | 0.1 | 1.5 | (0.6-4.1) | 0.6 | 1.3 | (0.4-4.3) | 0.2 | 1.0 | (0.2-5.2) | 0.0 |
|  |  |  |  |  |  |  |  |  |  |  |  |  |  |  |  |  |  |  |

Abbreviations. MDD, major depressive disorder; OR, odds ratio; CI; confidence interval.

*Significant at the .05 level, two-sided test.

^1^Models are bivariate models with each demographic predictors in separate models, controlling for country dummies

| **Appendix Table S3 Bivariate predictors of effective coverage and its components among those with 12-month major depressive disorder, in all countries, among severe cases (n=1,244)^1^** | | | | | | | | | | | | | | | | | | |
| --- | --- | --- | --- | --- | --- | --- | --- | --- | --- | --- | --- | --- | --- | --- | --- | --- | --- | --- |
|  | | | | | | | | | | | | | | | | | | |
|  | **Among those with 12-month MDD severe cases (n=1,244), received contact coverage** | | | **Among those with 12-month MDD severe cases and contact coverage (n=644), received any pharmachotherapy** | | | **Among those with 12-month MDD severe cases and contact coverage (n=644), received adequate pharmacotherapy** | | | **Among those with 12-month MDD severe cases and contact coverage (n=644), received any psychotherapy** | | | **Among those with 12-month MDD severe cases and contact coverage (n=644), received adequate psychotherapy** | | | **Among those with 12-month MDD severe cases (n=1,244), received effective coverage** | | |
|  | **OR** | **(95% CI)** | **F test** | **OR** | **(95% CI)** | **F test** | **OR** | **(95% CI)** | **F test** | **OR** | **(95% CI)** | **F test** | **OR** | **(95% CI)** | **F test** | **OR** | **(95% CI)** | **F test** |
| Gender |  |  |  |  |  |  |  |  |  |  |  |  |  |  |  |  |  |  |
| Male | 1.2 | (0.8-1.6) | 0.9 | 0.5* | (0.3-0.8) | 6.8* | 0.8 | (0.5-1.4) | 0.4 | 1.0 | (0.6-1.6) | 0.1 | 0.9 | (0.6-1.5) | 0.1 | 0.8 | (0.5-1.5) | 0.4 |
| Female (Ref) | REF |  |  | REF |  |  | REF |  |  | REF |  |  | REF |  |  | REF |  |  |
| Age |  |  |  |  |  |  |  |  |  |  |  |  |  |  |  |  |  |  |
| 18-29 | 1.0 | (0.6-1.8) | 3.4* | 0.6 | (0.2-1.3) | 5.0* | 1.3 | (0.6-3.0) | 2.1 | 2.4* | (1.1-5.4) | 2.4 | 2.4* | (1.1-5.2) | 2.6 | 1.6 | (0.6-4.0) | 2.3 |
| 30-44 | 1.4 | (0.8-2.2) |  | 1.3 | (0.6-2.8) |  | 1.9 | (1.0-3.5) |  | 2.3* | (1.2-4.7) |  | 2.4* | (1.2-4.8) |  | 2.5* | (1.2-5.3) |  |
| 45-59 | 1.9* | (1.1-3.1) |  | 2.4* | (1.1-5.2) |  | 2.2* | (1.1-4.2) |  | 1.6 | (0.8-3.3) |  | 1.5 | (0.7-3.0) |  | 2.3* | (1.0-5.2) |  |
| 60+ (Ref) | REF |  |  | REF |  |  | REF |  |  | REF |  |  | REF |  |  | REF |  |  |
| Marital status |  |  |  |  |  |  |  |  |  |  |  |  |  |  |  |  |  |  |
| Separated, divorced, or widowed | 1.2 | (0.9-1.7) | 0.7 | 1.1 | (0.7-1.8) | 1.3 | 1.0 | (0.6-1.7) | 0.2 | 0.9 | (0.6-1.5) | 0.8 | 0.9 | (0.6-1.6) | 0.4 | 1.0 | (0.5-1.8) | 0.1 |
| Never married | 1.0 | (0.7-1.4) |  | 0.6 | (0.3-1.2) |  | 0.8 | (0.5-1.4) |  | 1.4 | (0.8-2.5) |  | 1.2 | (0.7-2.2) |  | 0.9 | (0.5-1.6) |  |
| Married or cohabiting (Ref) | REF |  |  | REF |  |  | REF |  |  | REF |  |  | REF |  |  | REF |  |  |
| Income |  |  |  |  |  |  |  |  |  |  |  |  |  |  |  |  |  |  |
| Low | 0.5* | (0.4-0.8) | 3.9* | 1.2 | (0.6-2.3) | 0.2 | 1.0 | (0.5-2.0) | 0.8 | 0.6 | (0.4-1.2) | 1.1 | 0.8 | (0.4-1.4) | 0.6 | 0.7 | (0.3-1.6) | 0.6 |
| Low-Average | 0.5* | (0.3-0.8) |  | 1.0 | (0.5-1.8) |  | 1.4 | (0.7-2.6) |  | 0.9 | (0.5-1.7) |  | 1.0 | (0.5-1.8) |  | 1.0 | (0.4-2.2) |  |
| Average-High | 0.5* | (0.3-0.8) |  | 1.2 | (0.6-2.3) |  | 1.5 | (0.7-2.9) |  | 1.0 | (0.5-1.9) |  | 1.1 | (0.6-2.2) |  | 1.2 | (0.5-2.6) |  |
| High (Ref) | REF |  |  | REF |  |  | REF |  |  | REF |  |  | REF |  |  | REF |  |  |
| Level of education |  |  |  |  |  |  |  |  |  |  |  |  |  |  |  |  |  |  |
| Low | 0.5* | (0.3-0.9) | 2.2 | 0.9 | (0.4-2.1) | 0.5 | 0.6 | (0.3-1.2) | 1.0 | 0.5* | (0.3-1.0) | 1.6 | 0.6 | (0.3-1.1) | 1.4 | 0.4* | (0.2-0.9) | 1.9 |
| Low-Average | 0.6* | (0.4-0.9) |  | 1.1 | (0.5-2.3) |  | 0.6 | (0.3-1.3) |  | 0.6 | (0.3-1.2) |  | 0.6 | (0.3-1.2) |  | 0.5 | (0.3-1.1) |  |
| Average-High | 0.7 | (0.4-1.2) |  | 1.4 | (0.6-2.9) |  | 0.9 | (0.4-1.9) |  | 0.7 | (0.4-1.4) |  | 0.8 | (0.4-1.6) |  | 0.6 | (0.3-1.2) |  |
| High (Ref) | REF |  |  | REF |  |  | REF |  |  | REF |  |  | REF |  |  | REF |  |  |
| Type of insurance |  |  |  |  |  |  |  |  |  |  |  |  |  |  |  |  |  |  |
| None (Ref) | REF |  |  | REF |  |  | REF |  |  | REF |  |  | REF |  |  | REF |  |  |
| Direct Private/Optional Insurance | 2.1* | (1.0-4.2) | 2.1 | 1.8 | (0.7-5.1) | 0.8 | 1.1 | (0.4-3.0) | 0.2 | 1.7 | (0.7-4.3) | 2.0 | 2.4 | (1.0-5.8) | 2.5 | 2.1 | (0.6-6.8) | 0.8 |
| Any other types of insurance | 1.5 | (0.9-2.4) |  | 1.2 | (0.5-2.8) |  | 1.2 | (0.6-2.8) |  | 0.9 | (0.4-1.7) |  | 1.1 | (0.6-2.2) |  | 1.8 |  |  |
| Insurance |  |  |  |  |  |  |  |  |  |  |  |  |  |  |  |  |  |  |
| Direct Private/Optional Insurance (Yes) | 1.5 | (0.9-2.6) | 2.6 | 1.6 | (0.8-3.2) | 1.4 | 0.9 | (0.4-1.8) | 0.2 | 1.9 | (1.0-3.8) | 3.6 | 2.1* | (1.1-4.1) | 5.1* | 1.2 | (0.6-2.4) | 0.2 |
| **Appendix Table S3 continued. Bivariate predictors of effective coverage and its components among those with 12-month major depressive disorder, in all countries, among severe cases (n=1,244)^1^** | | | | | | | | | | | | | | | | | | |
|  |  | | |  | | |  | | |  | | |  | | |  | | |
|  | **Among those with 12-month MDD severe cases (n=1,244), received contact coverage** | | | **Among those with 12-month MDD severe cases and contact coverage (n=644), received any pharmachotherapy** | | | **Among those with 12-month MDD severe cases and contact coverage (n=644), received adequate pharmacotherapy** | | | **Among those with 12-month MDD severe cases and contact coverage (n=644), received any psychotherapy** | | | **Among those with 12-month MDD severe cases and contact coverage (n=644), received adequate psychotherapy** | | | **Among those with 12-month MDD severe cases (n=1,244), received effective coverage** | | |
|  | **OR** | **(95% CI)** | **F test** | **OR** | **(95% CI)** | **F test** | **OR** | **(95% CI)** | **F test** | **OR** | **(95% CI)** | **F test** | **OR** | **(95% CI)** | **F test** | **OR** | **(95% CI)** | **F test** |
| Employment status |  |  |  |  |  |  |  |  |  |  |  |  |  |  |  |  |  |  |
| Homemaker | 1.0 | (0.6-1.5) | 0.6 | 1.7 | (0.8-3.5) | 1.6 | 1.4 | (0.7-2.6) | 1.6 | 1.0 | (0.5-1.8) | 1.1 | 0.9 | (0.5-1.8) | 1.8 | 1.0 | (0.4-2.1) | 1.7 |
| Other | 1.1 | (0.7-1.6) |  | 1.1 | (0.6-2.2) |  | 1.9* | (1.1-3.3) |  | 1.6 | (1.0-2.5) |  | 1.7* | (1.0-2.9) |  | 2.0* | (1.1-3.7) |  |
| Retired | 0.9 | (0.5-1.6) |  | 1.4 | (0.6-3.4) |  | 1.6 | (0.7-3.5) |  | 1.0 | (0.5-2.0) |  | 1.0 | (0.6-2.0) |  | 1.2 | (0.5-2.7) |  |
| Student | 2.5 | (0.7-8.6) |  | 0.4 | (0.1-1.3) |  | 1.7 | (0.4-7.8) |  | 2.2 | (0.6-8.2) |  | 2.8 | (0.7-10.9) |  | 2.4 | (0.4-14.1) |  |
| Working (Ref) | REF |  |  | REF |  |  | REF |  |  | REF |  |  | REF |  |  | REF |  |  |
| Survey Year |  |  |  |  |  |  |  |  |  |  |  |  |  |  |  |  |  |  |
| Continuous | 1.0 | (0.7-1.5) | 0.1 | 1.3 | (0.7-2.1) | 0.7 | 1.3 | (0.8-2.2) | 1.0 | 1.7* | (1.1-2.7) | 5.0* | 1.5 | (0.9-2.5) | 2.3 | 1.3 | (0.6-2.8) | 0.5 |
|  |  |  |  |  |  |  |  |  |  |  |  |  |  |  |  |  |  |  |

Abbreviations. MDD, major depressive disorder; OR, odds ratio; CI; confidence interval.

*Significant at the .05 level, two-sided test.

^1^Models are bivariate models with each demographic predictors in separate models, controlling for country dummies.

| **Appendix Table S4. Bivariate predictors of effective coverage and its components among those with 12-month major depressive disorder, in high income countries, among severe cases (n=730)^1^** | | | | | | | | | | | | | | | | | | |
| --- | --- | --- | --- | --- | --- | --- | --- | --- | --- | --- | --- | --- | --- | --- | --- | --- | --- | --- |
|  | | | | | | | | | | | | | | | | | | |
|  | **Among those with 12-month MDD severe cases (n=730), received contact coverage** | | | **Among those with 12-month MDD severe cases and contact coverage (n=464), received any pharmachotherapy** | | | **Among those with 12-month MDD severe cases and contact coverage (n=464), received adequate pharmacotherapy** | | | **Among those with 12-month MDD severe cases and contact coverage (n=464), received any psychotherapy** | | | **Among those with 12-month MDD severe cases and contact coverage (n=464), received adequate psychotherapy** | | | **Among those with 12-month MDD severe cases (n=730), received effective coverage** | | |
|  | **OR** | **(95% CI)** | **F test** | **OR** | **(95% CI)** | **F test** | **OR** | **(95% CI)** | **F test** | **OR** | **(95% CI)** | **F test** | **OR** | **(95% CI)** | **F test** | **OR** | **(95% CI)** | **F test** |
| Gender |  |  |  |  |  |  |  |  |  |  |  |  |  |  |  |  |  |  |
| Male | 0.9 | (0.6-1.3) | 0.3 | 0.7 | (0.3-1.4) | 1.1 | 0.9 | (0.5-1.7) | 0.1 | 1.2 | (0.6-2.0) | 0.2 | 1.0 | (0.6-1.7) | 0.0 | 0.8 | (0.4-1.5) | 0.6 |
| Female (Ref) | REF |  |  | REF |  |  | REF |  |  | REF |  |  | REF |  |  | REF |  |  |
| Age |  |  |  |  |  |  |  |  |  |  |  |  |  |  |  |  |  |  |
| 18-29 | 1.1 | (0.5-2.2) | 4.1* | 0.4 | (0.2-1.3) | 3.5* | 0.9 | (0.3-2.3) | 1.2 | 2.4* | (1.0-5.7) | 1.5 | 1.7 | (0.8-3.9) | 0.8 | 1.0 | (0.3-3.1) | 1.5 |
| 30-44 | 1.7 | (1.0-3.0) |  | 0.8 | (0.3-2.2) |  | 1.3 | (0.6-2.6) |  | 2.0 | (0.9-4.3) |  | 1.8 | (0.8-3.8) |  | 1.8 | (0.8-3.9) |  |
| 45-59 | 2.3* | (1.2-4.2) |  | 2.2 | (0.8-6.0) |  | 1.8 | (0.8-3.7) |  | 1.7 | (0.8-3.8) |  | 1.4 | (0.7-3.1) |  | 2.0 | (0.9-4.5) |  |
| 60+ (Ref) | REF |  |  | REF |  |  | REF |  |  | REF |  |  | REF |  |  | REF |  |  |
| Marial status |  |  |  |  |  |  |  |  |  |  |  |  |  |  |  |  |  |  |
| Separated, divorced, or widowed | 1.3 | (0.8-1.9) | 0.6 | 0.8 | (0.4-1.4) | 1.1 | 0.7 | (0.4-1.3) | 1.6 | 0.7 | (0.4-1.2) | 0.8 | 0.7 | (0.4-1.3) | 0.8 | 0.7 | (0.4-1.4) | 1.2 |
| Never married | 1.0 | (0.6-1.6) |  | 0.6 | (0.3-1.2) |  | 0.6 | (0.3-1.2) |  | 0.9 | (0.4-1.7) |  | 0.8 | (0.4-1.4) |  | 0.6 | (0.3-1.3) |  |
| Married or cohabiting (Ref) | REF |  |  | REF |  |  | REF |  |  | REF |  |  | REF |  |  | REF |  |  |
| Income |  |  |  |  |  |  |  |  |  |  |  |  |  |  |  |  |  |  |
| Low | 0.7 | (0.4-1.3) | 1.1 | 0.7 | (0.3-1.6) | 0.5 | 1.3 | (0.6-2.5) | 1.1 | 0.7 | (0.4-1.4) | 1.0 | 0.8 | (0.4-1.5) | 1.0 | 0.9 | (0.4-2.2) | 0.4 |
| Low-Average | 0.7 | (0.4-1.2) |  | 0.8 | (0.4-1.7) |  | 1.5 | (0.8-2.8) |  | 1.1 | (0.5-2.2) |  | 1.2 | (0.6-2.6) |  | 1.2 | (0.5-2.6) |  |
| Average-High | 0.6 | (0.3-1.1) |  | 1.1 | (0.5-2.6) |  | 1.8 | (0.9-3.5) |  | 1.2 | (0.6-2.6) |  | 1.3 | (0.6-2.9) |  | 1.3 | (0.6-3.2) |  |
| High (Ref) | REF |  |  | REF |  |  | REF |  |  | REF |  |  | REF |  |  | REF |  |  |
| Level of education |  |  |  |  |  |  |  |  |  |  |  |  |  |  |  |  |  |  |
| Low | 0.6 | (0.3-1.2) | 0.9 | 0.6 | (0.2-2.0) | 0.7 | 0.8 | (0.4-1.6) | 0.9 | 0.6 | (0.3-1.2) | 1.0 | 0.6 | (0.3-1.4) | 0.8 | 0.5 | (0.2-1.2) | 1.1 |
| Low-Average | 0.8 | (0.4-1.6) |  | 0.6 | (0.2-1.6) |  | 0.6 | (0.3-1.4) |  | 0.7 | (0.3-1.5) |  | 0.7 | (0.3-1.4) |  | 0.6 | (0.3-1.2) |  |
| Average-High | 0.7 | (0.3-1.3) |  | 1.0 | (0.4-2.7) |  | 1.1 | (0.5-2.3) |  | 0.9 | (0.5-1.7) |  | 1.0 | (0.5-2.0) |  | 0.6 | (0.3-1.3) |  |
| High (Ref) | REF |  |  | REF |  |  | REF |  |  | REF |  |  | REF |  |  | REF |  |  |
| Type of insurance |  |  |  |  |  |  |  |  |  |  |  |  |  |  |  |  |  |  |
| None (Ref) | REF |  |  | REF |  |  | REF |  |  | REF |  |  | REF |  |  | REF |  |  |
| Direct Private/Optional Insurance | 1.3 | (0.6-3.0) | 1.9 | 5.6* | (1.3-23.1) | 3.2* | 1.4 | (0.4-4.6) | 0.6 | 1.1 | (0.4-3.0) | 0.2 | 1.6 | (0.6-4.3) | 0.6 | 2.4 | (0.6-10.0) | 1.2 |

| **Appendix Table S4 continued. Bivariate predictors of effective coverage and its components among those with 12-month major depressive disorder, in high income countries, among severe cases (n=730)^1^** | | | | | | | | | | | | | | | | | | | |
| --- | --- | --- | --- | --- | --- | --- | --- | --- | --- | --- | --- | --- | --- | --- | --- | --- | --- | --- | --- |
|  |  | | |  | | |  | | |  | | | |  | | |  | | |
|  | **Among those with 12-month MDD severe cases (n=730), received contact coverage** | | | **Among those with 12-month MDD severe cases and contact coverage (n=464), received any pharmachotherapy** | | | **Among those with 12-month MDD severe cases and contact coverage (n=464), received adequate pharmacotherapy** | | | **Among those with 12-month MDD severe cases and contact coverage (n=464), received any psychotherapy** | | | | **Among those with 12-month MDD severe cases and contact coverage (n=464), received adequate psychotherapy** | | | **Among those with 12-month MDD severe cases (n=730), received effective coverage** | | |
|  | **OR** | **(95% CI)** | **F test** | **OR** | **(95% CI)** | **F test** | **OR** | **(95% CI)** | **F test** | **OR** | **(95% CI)** | **F test** | | **OR** | **(95% CI)** | **F test** | **OR** | **(95% CI)** | **F test** |
| Any other types of insurance | 1.7 | (1.0-3.1) |  | 2.0 | (0.9-4.4) |  | 1.6 | (0.6-4.2) |  | 0.9 | (0.4-1.8) |  | | 1.1 | (0.5-2.2) |  | 2.5 | (0.8-7.9) |  |
| Direct Private/Optional Insurance (Yes) | 0.8 | (0.5-1.5) | 0.3 | 3.2 | (0.9-11.3) | 3.5 | 0.9 | (0.4-2.0) | 0.2 | 1.3 | (0.6-2.7) | 0.4 | | 1.5 | (0.7-3.1) | 1.1 | 1.1 | (0.5-2.3) | 0.1 |
| Employment status |  |  |  |  |  |  |  |  |  |  |  |  | |  |  |  |  |  |  |
| Homemaker | 0.9 | (0.5-1.7) | 0.5 | 1.2 | (0.5-2.7) | 0.2 | 1.8 | (0.8-4.2) | 1.4 | 1.6 | (0.7-3.8) | 1.8 | | 1.9 | (0.8-4.6) | 2.4 | 1.7 | (0.6-4.3) | 2.0 |
| Other | 1.2 | (0.7-2.1) |  | 1.0 | (0.4-2.3) |  | 1.8* | (1.0-3.2) |  | 1.7 | (1.0-2.9) |  | | 1.8* | (1.0-3.2) |  | 2.1* | (1.1-4.1) |  |
| Retired | 0.8 | (0.4-1.4) |  | 1.0 | (0.4-2.7) |  | 1.3 | (0.5-3.1) |  | 0.9 | (0.4-1.8) |  | | 1.1 | (0.5-2.2) |  | 1.0 | (0.4-2.5) |  |
| Student | 0.7 | (0.1-3.8) |  | 0.5 | (0.1-3.3) |  | 2.6 | (0.4-18.0) |  | 5.7 | (0.7-44.2) | |  | 8.7* | (1.1-71.3) |  | 3.7 | (0.6-23.4) |  |
| Working (Ref) | REF |  |  | REF |  |  | REF |  |  | REF |  |  | | REF |  |  | REF |  |  |
| Survey year |  |  |  |  |  |  |  |  |  |  |  |  | |  |  |  |  |  |  |
| Continuous | 1.3 | (0.8-2.1) | 1.2 | 1.7 | (1.0-3.0) | 3.5 | 1.4 | (0.8-2.4) | 1.1 | 1.5 | (0.9-2.7) | 2.2 | | 1.5 | (0.8-2.7) | 1.6 | 1.5 | (0.7-3.3) | 1.2 |
|  |  |  |  |  |  |  |  |  |  |  |  |  | |  |  |  |  |  |  |

Abbreviations. MDD, major depressive disorder; OR, odds ratio; CI; confidence interval.

*Significant at the .05 level, two-sided test.

^1^Models are bivariate models with each demographic predictors in separate models, controlling for country dummies.

| **Appendix Table S5. Bivariate predictors of effective coverage and its components among those with 12-month major depressive disorder, in low and middle countries, among severe cases (n=514)^1^** | | | | | | | | | | | | | | | | | | |
| --- | --- | --- | --- | --- | --- | --- | --- | --- | --- | --- | --- | --- | --- | --- | --- | --- | --- | --- |
|  |  | | |  | | |  | | |  | | |  | | |  | | |
|  | **Among those with 12-month MDD severe cases (n=514), received contact coverage** | | | **Among those with 12-month MDD severe cases and contact coverage (n=180), received any pharmachotherapy** | | | **Among those with 12-month MDD severe cases and contact coverage (n=180), received adequate pharmacotherapy** | | | **Among those with 12-month MDD severe cases and contact coverage (n=180), received any psychotherapy** | | | **Among those with 12-month MDD severe cases and contact coverage (n=180), received adequate psychotherapy** | | | **Among those with 12-month MDD severe cases (n=514), received effective coverage** | | |
|  | **OR** | **(95% CI)** | **F test** | **OR** | **(95% CI)** | **F test** | **OR** | **(95% CI)** | **F test** | **OR** | **(95% CI)** | **F test** | **OR** | **(95% CI)** | **F test** | **OR** | **(95% CI)** | **F test** |
| Gender |  |  |  |  |  |  |  |  |  |  |  |  |  |  |  |  |  |  |
| Male | 1.7* | (1.1-2.6) | 5.6* | 0.3* | (0.1-0.6) | 10.0* | 0.6 | (0.2-1.8) | 0.8 | 0.6 | (0.2-1.6) | 0.9 | 0.8 | (0.3-2.0) | 0.3 | 1.0 | (0.3-3.7) | 0.0 |
| Female (Ref) | REF |  |  | REF |  |  | REF |  |  | REF |  |  | REF |  |  | REF |  |  |
| Age |  |  |  |  |  |  |  |  |  |  |  |  |  |  |  |  |  |  |
| Continuous | 1.0 | (1.0-1.0) | 1.5 | 1.0 | (1.0-1.1) | 2.0 | 1.0 | (1.0-1.0) | 0.6 | 1.0 | (0.9-1.0) | 1.3 | 1.0* | (0.9-1.0) | 5.6* | 1.0 | (1.0-1.0) | 1.7 |
| Marital status |  |  |  |  |  |  |  |  |  |  |  |  |  |  |  |  |  |  |
| Separated, divorced, or widowed | 1.2 | (0.6-2.0) | 0.2 | 2.0 | (0.8-5.6) | 1.3 | 2.8* | (1.1-7.3) | 3.4* | 1.9 | (0.8-4.7) | 3.8* | 1.7 | (0.6-4.8) | 3.1* | 2.4 | (0.7-8.0) | 2.1 |
| Never married | 0.9 | (0.5-1.7) |  | 0.7 | (0.2-2.2) |  | 2.0 | (0.7-5.6) |  | 4.1* | (1.4-12.2) |  | 3.8* | (1.3-11.5) |  | 2.2 | (0.8-6.4) |  |
| Married or cohabiting (Ref) | REF |  |  | REF |  |  | REF |  |  | REF |  |  | REF |  |  | REF |  |  |
| Income |  |  |  |  |  |  |  |  |  |  |  |  |  |  |  |  |  |  |
| Low | 0.4* | (0.2-0.8) | 3.3* | 3.1* | (1.1-8.7) | 2.2 | 0.6 | (0.1-2.8) | 0.5 | 0.6 | (0.2-1.9) | 0.3 | 0.9 | (0.2-3.0) | 0.4 | 0.4 | (0.1-2.5) | 0.4 |
| Low-Average | 0.5* | (0.2-0.9) |  | 0.9 | (0.3-2.9) |  | 1.4 | (0.3-6.3) |  | 0.7 | (0.2-2.3) |  | 0.5 | (0.2-1.8) |  | 0.8 | (0.1-4.8) |  |
| Average-High | 0.5* | (0.2-0.9) |  | 0.9 | (0.2-3.1) |  | 1.0 | (0.2-5.2) |  | 0.6 | (0.2-2.2) |  | 0.8 | (0.2-2.8) |  | 0.9 | (0.2-4.8) |  |
| High (Ref) | REF |  |  | REF |  |  | REF |  |  | REF |  |  | REF |  |  | REF |  |  |
| Level of education |  |  |  |  |  |  |  |  |  |  |  |  |  |  |  |  |  |  |
| Low | 0.5 | (0.2-1.0) | 3.3* | 1.4 | (0.4-4.2) | 0.6 | 0.3* | (0.1-1.0) | 1.4 | 0.4 | (0.1-1.4) | 0.7 | 0.4 | (0.1-1.3) | 0.9 | 0.1* | (0.0-0.9) | 1.7 |
| Low-Average | 0.4* | (0.2-0.7) |  | 2.6 | (0.6-11.6) |  | 0.8 | (0.2-3.7) |  | 0.5 | (0.1-1.9) |  | 0.4 | (0.1-1.7) |  | 0.5 | (0.1-2.3) |  |
| Average-High | 0.7 | (0.3-1.5) |  | 1.9 | (0.5-6.7) |  | 0.6 | (0.1-3.4) |  | 0.5 | (0.1-2.2) |  | 0.5 | (0.1-2.3) |  | 0.5 | (0.1-2.7) |  |
| High (Ref) | REF |  |  | REF |  |  | REF |  |  | REF |  |  | REF |  |  | REF |  |  |
| Insurance |  |  |  |  |  |  |  |  |  |  |  |  |  |  |  |  |  |  |
| Direct Private/Optional Insurance (Yes) | 3.6* | (1.5-8.5) | 8.6* | 0.8 | (0.4-2.0) | 0.2 | 0.9 | (0.2-3.6) | 0.0 | 3.8* | (1.0-14.2) | 4.0* | 3.7* | (1.1-12.3) | 4.5* | 1.6 | (0.4-7.0) | 0.4 |
| Employment status |  |  |  |  |  |  |  |  |  |  |  |  |  |  |  |  |  |  |
| Working (Yes) | 0.9 | (0.6-1.4) | 0.2 | 0.6 | (0.3-1.5) | 1.0 | 0.5 | (0.2-1.4) | 1.6 | 1.1 | (0.5-2.4) | 0.0 | 1.3 | (0.6-2.9) | 0.3 | 0.9 | (0.3-3.2) | 0.0 |
| Survey year |  |  |  |  |  |  |  |  |  |  |  |  |  |  |  |  |  |  |
| Continuous | 0.6 | (0.3-1.4) | 1.3 | 0.6 | (0.2-2.3) | 0.5 | 1.1 | (0.4-3.2) | 0.0 | 2.3 | (1.0-5.4) | 3.6 | 1.5 | (0.6-3.7) | 0.8 | 0.7 | (0.1-3.1) | 0.3 |
|  |  |  |  |  |  |  |  |  |  |  |  |  |  |  |  |  |  |  |

Abbreviations. MDD, major depressive disorder.

*Significant at the .05 level, two-sided test

^1^Models are bivariate models with each demographic predictors in separate models, controlling for country dummies.

| **Appendix Box S1 Antidepressants and classes** | |  |
| --- | --- | --- |
| **Drug name** | **Classification** |  |
| Bupropion | NDRI |  |
| Duloxetine | SNRI |  |
| Medifoxamine | SDRI |  |
| Milnacipran | SNRI |  |
| Mirtazapine | NaSSA |  |
| Moclobemide | MAOI |  |
| Nefazodone | SARI |  |
| Reboxetine | NRI |  |
| Venlafaxine | SNRI |  |
| Viloxazine | NRI |  |
| Citalopram | SSRI |  |
| Escitalopram | SSRI |  |
| Fluoxetine | SSRI |  |
| Fluvoxamine | SSRI |  |
| Paroxetine | SSRI |  |
| Sertraline | SSRI |  |
| Amineptine | TCA |  |
| Amitriptyline | TCA |  |
| Amoxapine | TCA |  |
| Butriptyline | TCA |  |
| Clomipramine | TCA |  |
| Desipramine | TCA |  |
| Dibenzepin | TCA |  |
| Dothiepin | TCA |  |
| Doxepin | TCA |  |
| Imipramine | TCA |  |
| Lofepramine | TCA |  |
| Maprotiline | TCA |  |
| Melitracen | TCA |  |
| Mianserin | TCA |  |
| Nortriptyline | TCA |  |
| Opipramol | TCA |  |
| Protriptyline | TCA |  |
| Tianeptine | TCA |  |
| Trimipramine | TCA |  |
| Isocarboxazid | MAOI |  |
| Phenelzine | MAOI |  |
| St. John's Wort | Herbs |  |
| Tranylcypromi | MAOI |  |
| Trazodone | SARI |  |

Abbreviations: AD, Antidepressant; MAOI, Monoamine oxidase inhibitors; NaSSA, Noradrenergic and specific serotonergic antidepressant; NDRI, Norepinephrine–dopamine reuptake inhibitor; NRI, Norepinephrine reuptake inhibitor; SARI, Serotonin antagonist and reuptake inhibitor; SNRI, Serotonin and norepinephrine reuptake inhibitors; SSRI, Selective serotonin reuptake inhibitors; TCA, Tricyclic antidepressants.
